# A comparative analysis of approaches to network-dismantling

**DOI:** 10.1038/s41598-018-31902-8

**Published:** 2018-09-10

**Authors:** Sebastian Wandelt, Xiaoqian Sun, Daozhong Feng, Massimiliano Zanin, Shlomo Havlin

**Affiliations:** 1 National Key Laboratory of CNS/ATM, School of Electronic and Information Engineering, Beihang University, 100191 Beijing, China; 2 National Engineering Laboratory of Multi-Modal Transportation Big Data, 100191 Beijing, China; 3 Beijing Advanced Innovation Center for Big Data-based Precision Medicine, Beihang University, 100083 Beijing, China; 4 Centro de Tecnologica Biomedica, Universidad Politecnica de Madrid, 28223 Madrid, Spain; 5 Faculdade de Ciecias e Tecnologia, Universidade Nova de Lisboa, 2829-516 Caparica, Portugal; 6 Department of Physics, Bar-Ilan University, Ramat-Gan, 52900 Israel

## Abstract

Estimating, understanding, and improving the robustness of networks has many application areas such as bioinformatics, transportation, or computational linguistics. Accordingly, with the rise of network science for modeling complex systems, many methods for robustness estimation and network dismantling have been developed and applied to real-world problems. The state-of-the-art in this field is quite fuzzy, as results are published in various domain-specific venues and using different datasets. In this study, we report, to the best of our knowledge, on the analysis of the largest benchmark regarding network dismantling. We reimplemented and compared 13 competitors on 12 types of random networks, including ER, BA, and WS, with different network generation parameters. We find that network metrics, proposed more than 20 years ago, are often non-dominating competitors, while many recently proposed techniques perform well only on specific network types. Besides the solution quality, we also investigate the execution time. Moreover, we analyze the similarity of competitors, as induced by their node rankings. We compare and validate our results on real-world networks. Our study is aimed to be a reference for selecting a network dismantling method for a given network, considering accuracy requirements and run time constraints.

## Introduction

During the last decades, empirical studies have characterized a plethora of real-world systems through the complex network perspective^1,2^, including air transport^3–7^, power grids^8,9^, the Internet backbone^10,11^, inter-bank^12^, or inter-personal networks^13^. One of the most relevant topics has been the assessment of their robustness, i.e. the capacity to keep performing their intended function after a major failure. This is not surprising, taking into account that all previous examples share a common feature: they are critical infrastructures, in that their failure would lead to major disruptions in our society. Examples of recent extensive, wide-ranging network failures include the European air traffic disruption caused by the Icelandic Eyjafallajökull volcano eruption^14^, large-scale power outages in the United States^15^, computer virus spreading^16^, or the cross-continental supply-chain shortages in the Japanese 2011 tsunami aftermath^17^, and others^18^. In all these events, the affected countries had to face extremely high economic costs^19^. Researchers have thus tried to quantify how the connectivity is affected by node (and link) removal, both due to random (unintentional) and targeted (intentional) processes. A complementary problem soon arose: the identification of the most effective strategies for disrupting (or attacking) a network. Such analysis yields important insights in a twofold way. First of all, it allows to move from assessing to improving resilience, by forecasting what a rational attacker might do and thus identifying which elements should prima facie be protected. Secondly, there are instances in which we actually need to disrupt a network, as for instance to stop the propagation of a disease or a computer virus, or to impair the growth of a cancer cell. In these situations, designing an efficient disruption strategy means achieving the goal while respectively minimizing the cost of immunisation strategies or the number of drugs to be prescribed.

Research on connectivity robustness has been performed in various scientific disciplines, the most important ones including complex network theory, bioinformatics, transportation/logistics and communication. While there are subtle differences in these robustness definitions, the goal is always to identify the most critical nodes in a given network, i.e. those whose removal would severely impair the network dynamics. Notably, complex network theory has allowed to obtain some principle results that are independent from the specific system under study. Most networks, sharing a scale-free structure, present a well-recognized resilience against random failures^20^, but disintegrate rapidly under intentional attacks primarily targeting important nodes^21–23^. Moreover, initial shocks can sometimes lead to cascading failures^24^.

In parallel to those theoretical results, several methods have been proposed in the last decade for dismantling a network, i.e. for identifying the sequence of nodes that maximizes the damage on the network connectivity. As the exact solution is computationally intractable for medium and large networks, several approximations have been proposed, based on collective influence^25^, decycling and tree breaking^26^, articulation points^27^, spectrality^28^, or network communities^29^. Other related works rely on standard network metrics and their variants, including degree, k-shell decomposition^30^, betweenness^31^, and approximate betweenness^32^.

In spite of these results, two major problems are subject to further research. First of all, the proposed methods substantially differ in terms of underlying principles, performance and computational cost. Some of them are more efficient in dismantling specific types of networks; others have a general applicability, but the scaling of their computational cost reduces their usefulness in large systems. Although newly published methods are sometimes compared to prior works, the selection of these latter ones is largely arbitrary and comparisons are carried out on few distinct networks. Secondly, even if such results are reported, their interpretation is usually far from trivial, as there is no theory supporting the selection of the best metric for measuring (and hence compare) algorithm’s performances. As a consequence of the heterogeneity of approaches and problems, the lack of common benchmarks, and the dispersal of research in different communities, today it is hardly possible to choose the best algorithm for a given problem.

In this study, we present (to the best of our knowledge) the most comprehensive benchmark on network dismantling algorithms to date. We have (re)implemented a set of 13 competitors, to ensure code homogeneity; and have tested them on a large set of networks of different topologies and sizes. We identified large heterogeneities in algorithm performances, as well as differences in run time of factors of more than 1000 between the fastest and slowest algorithms. These results allow us to draw several interesting conclusions about optimality, scalability, applicability, as well as potential future research directions. Additionally, the practitioner interested in selecting an algorithm for network dismantling and with a clear application in mind, will here find a valuable guide for making an informed choice.

The remainder of this paper is organized as follows. We describe the benchmark setup, the networks, and competitors in Section 2. All evaluation results are presented and discussed in Section 3. The paper is concluded with Section 4.

## Methods

### Measuring attack efficacy

Inspired by the well-known concept of percolation in statistical physics^33–37^, the robustness of a network is usually defined as the critical fraction of nodes that, when removed, causes a sudden disintegration^21^. The disintegration is measured as the relative reduction in the size of the giant (largest connected) component. The smaller the size of the remaining giant component, the more the network is considered to have been disintegrated^38^, the rationale being that the functionality of a network strongly depends on the number of connected nodes.

In this study, we use the robustness measure *R*^39^. Given a network composed of *N* nodes, *R* is defined as

1 $$R=\frac{1}{N}\sum _{Q=1}^{N}\,s(Q),$$

where *s*(*Q*) is the size of the giant component after removing *Q* nodes. Intuitively this is equivalent to assessing how many nodes the giant component contains when a new node is deleted from the network, and sum this for all nodes. Note that the computation of *R* requires a ranking of the nodes, defining the sequence in which they are deleted from the network. In general, we are interested in the minimum *R* over all possible node orders. Since the computation of this optimal node order is an NP hard problem, researchers resort to sub-optimal and approximated methods, either tailored specifically for network dismantling^25–28,40,41^, or based on traditional network metrics^30,31,42,43^.

**Figure 1:**
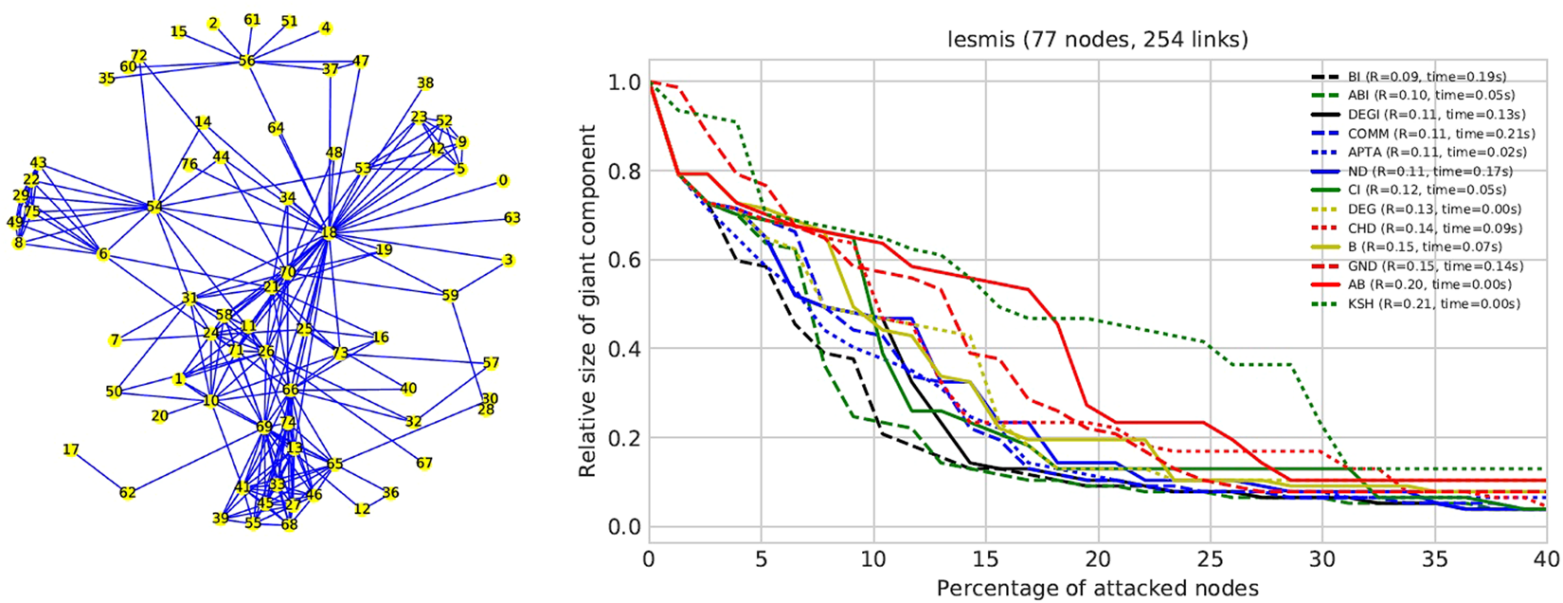
Example network (left) and its robustness curves (right). The network lesmis consists of 77 nodes and 254 links. The average degree is 6.597. The competitors in our study reveal very distinct attacking strategies and percolation thresholds for the network. In the legend, all methods are ordered by increasing value of R, see Eq. (1). The range of R is between 0.09 and 0.21, which shows the importance of selecting an appropriate attacking strategy for a given network.

In order to illustrate the problems that can arise from the use of sub-optimal attack algorithms, we here briefly present the results obtained for the *lesmis* network^44^. *Lesmis* encodes coappearances in the novel Les Miserables, is composed of 77 nodes and 254 links, and is frequently used in studies on complex networks. The network is depicted in Fig. 1(left), while its disruption process is shown in Fig. 1(right). In the ladder graph, each curve represents the evolution of the giant component size as more nodes are deleted, for one of the 13 algorithms here considered (see Section 2.2 for details); the legend also reports the corresponding *R* values and computation times. It can be seen that results are quite heterogeneous, suggesting that these methods rely on idiosyncratic strategies for defining the importance of nodes. Moreover, *R* values are spread over a large interval, from 0.09 (for BI, ABI) to 0.21 (for KSH): the best algorithms are thus twice as effective in dismantling the network than the worst one.

If one is usually interested in the strongest attack, and thus in the smallest *R* value, two issues have to be taken into account. First, even though BI and ABI have yielded the best results, this does not imply that these two algorithms will always outperform the others, as their performance is problem-dependent. Second, the low *R* value has to be balanced with the running time of the methods. This is especially relevant when designing attacks on larger networks, because the worst-case runtime complexity prohibits execution in a reasonable amount of time.

It is finally worth noting that the R value is a standard measure used in several recent studies, which aggregates the robustness over all nodes. The size of the giant component as a fraction of network size, on the other hand, requires to fix a specific attack length, e.g. 5%, 10% or 50% of the nodes. Alternatively, one can fix an interesting component size threshold and report the required length of an attack. In general, dismantling methods which perform poorly when considering a component threshold, will also perform poorly when assessed through R.

### Competitors

Below, we list the 13 methods evaluated and compared in our study. The first five are specifically designed for dismantling networks, while the remaining seven leverage on network metrics often used in empirical studies. Note that there are other methods, which are not used frequently, given that they are significantly harder to implement^45^.

- **ND**: Network dismantling (ND) assumes that for a large class of random graphs, the problem of network dismantling is connected to the decycling problem, which addressed the removal of vertices until a graph becomes acyclic^26^. The authors propose a three-stage Min-Sum algorithm for efficiently dismantling networks, which are summarized as follows. Firstly, at the core of the algorithm is a variant of Min-Sum message passing for decycling, developed in^41,46^. The second step has the goal of tree breaking. After all cycles are broken, some of the tree components may still be larger than the desired threshold. These components are further broken into smaller components, removing a fraction of nodes that vanishes in the large size limit. Finally, cycles are closed greedily, in order to improve the efficiency of the algorithm with many short cycles.
- **CI**: Collective influence (CI) is a node importance measure for identifying influential spreaders in a network^25^. The authors noted that the problem of influence is tightly related to the problem of network dismantling, i.e., the removal of most influential nodes in a network often dismantles the network into many disconnected non-extensive components. The collective influence of a node is measured by the number of nodes within a given radius k, usually referred to as a k-ball of a node. Intuitively, this measure is an extension of degree metric to take into account neighbors at a distance of k. In hierarchical networks, the CI value can be easily computed in *O*(*N* * *logN*) time. Originally designed for efficiently attacking hierarchical networks, CI has now been used in several research studies on general graphs. In order to reduce the computational cost, a max heap data structure^40^ has been included in the implementation.
- **APTA**: Brute-force articulation point attack (APTA) targets articulation points in a network^27^. An articulation point (AP) is a node whose removal disconnects a network. All APs can be identified by performing a variant of depth-first search, starting from a random node in the network; see^47^ for a linear-time implementation. It is surprising that the linear-time algorithm does not only reveal all APs, but also gives an estimate of the component sizes after removal of each AP from the network. This yields an attacking strategy by greedily attacking the AP with the largest effect (i.e., smallest maximum size of resulting components). If a network instance does not have a AP, for instance, a circle graph, then we attack one node with the highest-degree randomly. The resulting attacking method scales very well with the number of nodes, given the identification of node candidates in linear-time. Nevertheless, the greedy character of APTA should be understood: At each step of an attack, a locally-optimal AP is chosen, but there is no guarantee for a global optimum.
- **GND**: Generalized network dismantling (GND) was recently proposed as a method to dismantle networks while taking into account node-specific costs^28^. Under the assumption of unit costs for nodes, GND can be used for solving the standard network dismantling problem. GND relies on spectral cuts, using an efficient spectral approximation by a Power Laplacian operator, which can be computed in *O*(*N* * (*log*^2 + *ε*^*N*)), with *ε* being larger than 0. The actual choice of the value is involved^28^ (see the discussion in their supplementary^28^), but, essentially, any value larger than *ε* guarantees convergence. For the experiments in our study, we have set *ε* = 3. Larger values increased the computation time significantly, while not always improving the quality further.
- **COMM**: Community-based^29^ attacks initially rely on the identification of the communities composing the network, usually based on the concept of modularity^48^. In our study, communities are extracted through a general search strategy for the optimization of modularity^49^. Other community methods can be used, for instance, the widely-used Louvain method^50^. Given a community structure was obtained, inter-community connections are identified by calculating nodes’ betweenness centralities, and removed iteratively. For the remaining parts of the network, which cannot be divided into communities, nodes are ranked by degree.
- **DEG**: Degree is a simple local network metric, which quantifies the importance of a node by counting its number of direct neighbors. This indicator is simple and fast to compute, although it codifies no information about the macro-scale network structure. A static attack DEG is based on sorting nodes by descending degrees and removing them accordingly.
- **DEGI**: An iterative variant of DEG, introduced to account for dynamic changes in the degree of nodes while the attack is being executed^42^.
- **B**: Betweenness centrality^31,51^ is a macro-scale network metric measuring the number of times a node appears in the shortest path between pairs of nodes. Calculating the exact betweenness centrality entails a high computational cost, with a complexity of *O*(*nm*) when using Brandes’ algorithm^52^ on unweighted graphs, where *n* is the number of nodes and *m* is the number of edges in network.
- **BI**: An iterative variant of B, introduced to account for changes in the betweenness while nodes are being removed^42^.
- **AB**: Given the worst-case time complexity of betweenness (B) computation, it is often helpful to compute and use an approximate betweenness^32^. This algorithm reduces the time complexity by sampling only a subset of all possible node pairs in the network, such that only *logn* paths are taken into account.
- **ABI**: An iterative variant of AB, introduced to account for changes in the approximate betweenness when nodes are being removed^42^.
- **KSH**: K-shell iteration factor^53^ is based on the coreness of nodes in a network^30^. In general, a large value indicates that the node has a strong ability to spread information. The algorithm combines shell decomposition and iterative node removal, for then using changes in the neighbourhood as an estimator of the impact for each node.
- **CHD**: CoreHD attacks^54^ combine DEGI and k-core^30^ to achieve a decycling of networks. It iteratively removes the highest degree node among network 2-core graphs, until no 2-core graph remains, for then treating the remaining part through tree-breaking.

### Synthetic network models

In this study we initially evaluate all competitors against a set of synthetic networks - note that this evaluation will be extended to real-world networks in Section 3.5. Synthetic networks, i.e. networks that are the result of applying generative functions, present the advantage of displaying specific topological features that are both a priori known and tuneable.

**Figure 2:**
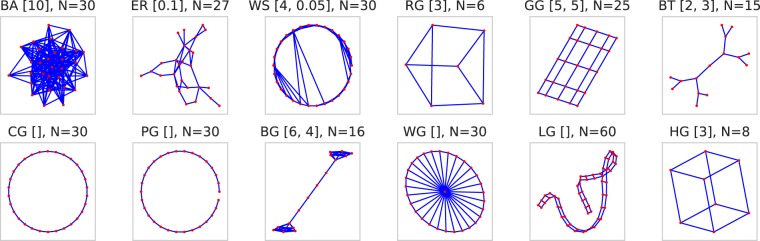
Visualization of one realization for each type of the 12 networks in this study. These realizations are to be understood as samples only, not all realizations of a network have highly similar visual properties, since their structure largely depends on the chosen parameters, particularly for BA, ER, WS, and RG.

For this study, we selected a collection of 12 network types, summarized in Table 1. Four of these types are standard complex network models: Barabasi-Albert (BA)^55^, Watt-Strogatz (WS)^56^, Erdos-Renyi (ER)^57^, and Regular Graphs (RG). The remaining eight types are specific graphs with interesting topologies or properties, making them valuable for robustness analyses: Circle Graphs (CG), Grid Graphs (GG), Path Graphs (PG), Barbell Graphs (BG), Wheel Graphs (WG), Ladder Graphs (LG), Binary Trees (BT), and Hyper Graphs (HG). Figure 2 visualizes one instance for each of the 12 networks types.

**Table 1:** Overview of 12 random network types.

ID	Name	Parameters (n = number of nodes)
BA	Barabasi-Albert	n in [100,500,1000], number of edges m per node in [1,3,5,7,9]
ER	Erdos-Renyi	n in [100,500,1000], edge probability p in [0.01,0.02,0.03,0.04,0.05]
WS	Watt-Strogatz	n in [100,500,1000], ring size k in [3,5,7,9], rewiring probability p = [0.1,0.4,0.7]
RG	Regular graphs	n in [100,500,1000], node degree d in [3,4,5]
GG	Grid graphs	Side length d in [5,10,15,20,25,30]
PG	Path graphs	n in [100,500,1000]
CG	Circle graphs	n in [100,500,1000]
WG	Wheel graphs	n in [100,500,1000]
LG	Ladder graphs	n in [100,500,1000]
BT	Binary trees	Branching factor r in [2,3], height of tree h in [3,4]
HG	Hypercube graphs	Dimension d in [2,3,4,5,6,7,8]
BG	Barbell graphs	Bell size m1 in [5,10,15,20], path length m2 in [5,10,15,20]

The generative functions supporting the creation of these synthetic networks include a set of parameters controlling the size and structure of the resulting network instances. For the sake of completeness, each synthetic model has been executed with several parameter combinations, each one of these including five random realizations. In total, results have been calculated over a set of 600 networks, representing a wide range of topological structures.

**Figure 3:**
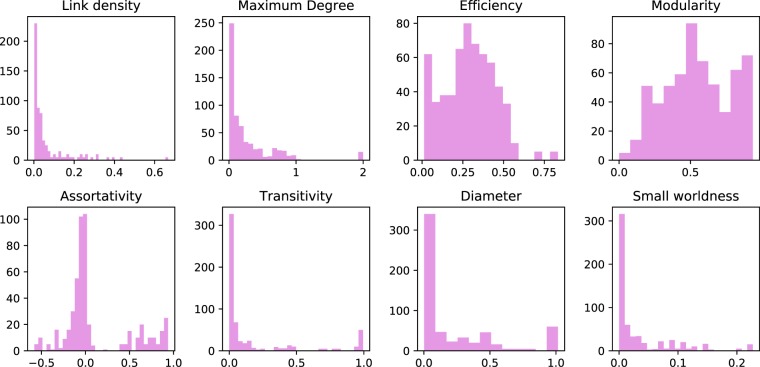
Statistics of selected topological metric values in the synthetic network set. The x-axis shows the range of values for each metric and the y-axis the absolute frequency of graphs having a metric value in that range.

**Figure 4:**
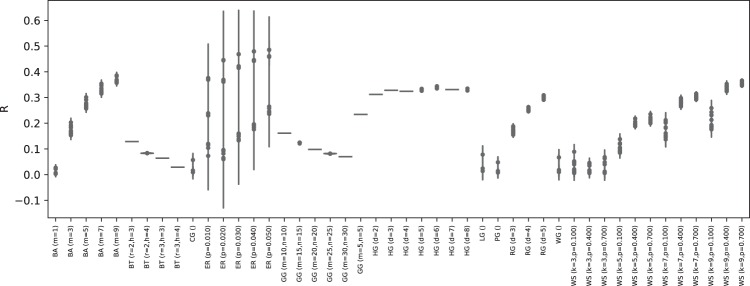
Minimum *R* values for all network sub-types in our study. It can be seen that, for each type, network realizations cover a wide range of R values, from highly fragile to very robust ones.

**Figure 5:**
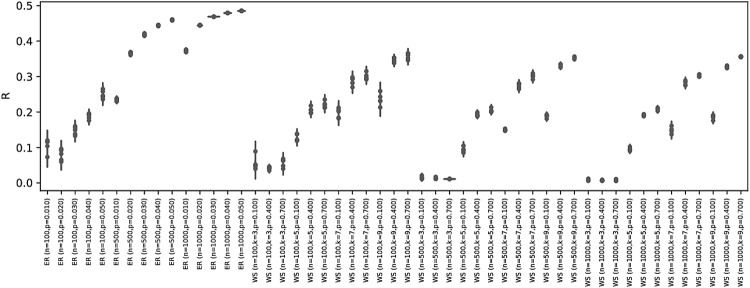
Minimum *R* values for two network sub-types ER and WS, with different network sizes. Each type-size combination covers a small range of *R* values.

We summarize eight of the most important topological properties of the synthetic networks in Fig. 3. The eight histograms confirm that the set is representative of many topological structures, including heterogeneous examples of modularity, efficiency and assortativity values. Additionally, Fig. 4 reports the *R* values of all networks, as obtained with the best competitor of those used in our study. Within each network type and network generation parameter group, networks have similar robustness properties, as highlighted by the small range of *R* values. The only exceptions are ER and WS, whose robustness heterogeneity depends largely on the interaction between the network size and generation parameters, i.e., the same parameter setting yields robust networks for small *n*, but highly vulnerable networks for larger *n*. In Fig. 5, we further split up the ER and WS networks by taking into account their size. It can be seen that with a fixed number of nodes, the range of *R* values is again reduced.

In synthesis, the set of synthetic networks considered is representative of very different topological structures, and of a wide range of robustness. This will ensure that subsequent analyses will not be biased by a specific network characteristic, and that results will have high generalizability.

## Results

In the following, we report the results of our study. Section 3.1 firstly compares all competitors with respect to their accuracy, i.e. how small is the obtained *R* value in each network type. The correlation between competitors, i.e. the relationship between the node rankings they yield, is analyzed in Section 3.2. In Section 3.3, we set the focus on the running time of each method and see how it changes with the size of the network. We combine both criteria, optimality and scalability, in Section 3.4, where we report results regarding the Pareto front of both dimensions, identifying dominating competitors. Section 3.5 compares the results obtained in Section 3.1–3.4 with those for real-world networks. Finally, we discuss the differences and similarities to other robustness estimation methods in Section 3.6 and additional results on modularity in Section 3.7. Code is available at https://github.com/hubsw/NetworkDismantling for free academic use.

### Ranking for attack efficacy

**Figure 6:**
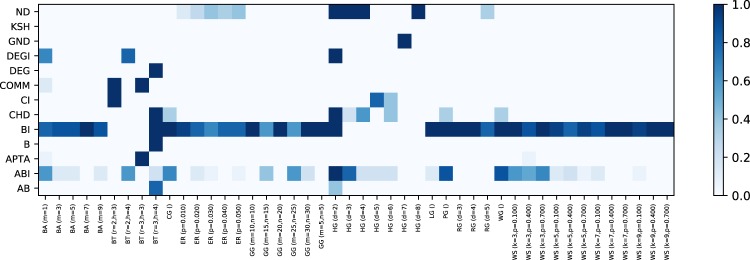
Relative frequency of results yielding minimum R values for each competitor and network subtype. BI is the competitor with the smallest R value, leading to the most damaging network attacks. Other, less competitive competitors include ABI and CHD. The performance of a method often depends on the network type: For instance, ND is excellent on several parameter combinations for hypercube graphs.

The major goal when estimating the robustness of a network is to compute the smallest valid *R*, where valid refers to the fact that it is calculated from a ranking over all nodes in the network. As we showed in Section 1, and particularly in Fig. 1, the *R* values yielded by different methods can substantially vary. Figure 6 visualizes the frequency that each competing method yields the smallest *R*, disaggregated by network type and generation parameters. We find that BI yields the best attack in 70−80% of the cases. The major exception is HG, whose network structure is better dismantled by using the CI technique. Next to BI, the two competitors ABI and CHD perform rather well on most networks in our study. On the other side of the ranking, the worst competitors are DEG, DEGI, KSH and COMM.

**Figure 7:**
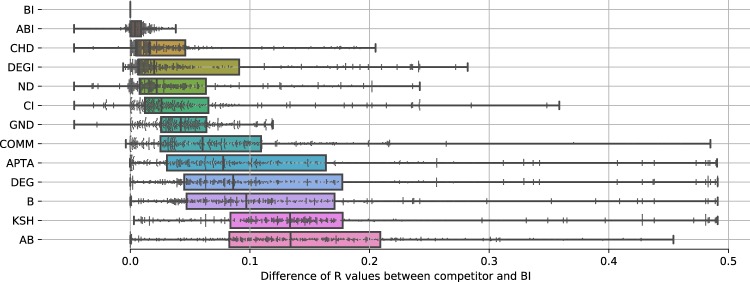
Comparison of R values for different competitors against BI, the most accurate method in our study. For each competitor, we plot the distribution of all R values as a box plot. Each box covers the values within boundaries X,Y. Competitors are sorted according to their median deviations to BI values. The best competitors are ABI and CHD, which have an absolute difference in R values of 0.01–0.02 for the majority of network instances. Moreover, their maximum deviation is between 0.05–0.08. Other competitors, including AB and DEG, have much higher deviations, with a difference of 0.5 as the maximum case, which essentially renders them useless for accurate estimations of network robustness.

Given that BI yields the smallest *R* for the majority of networks in our study, we here report on a comparison of the competitors’ performance using BI as a baseline. In Fig. 7, we report the difference betweeen the *R* value of BI for each competitor and network instance, with competitors sorted according to their respective median. The boxplot reveals that ABI and CHD are very close competitors to BI; nevertheless, while the median *R* difference is quite close to zero, differences in specific networks can reach 0.05 (ABI). On the other end of the ranking, the worst competitors, when BI is used as a baseline, are KSH and AB, with a median *R* difference of 0.13. Note that the worst algorithms in Fig. 7 do not need to (and indeed do not) coincide with those of Fig. 6, as both graphs represent different quantities. To illustrate, suppose a method that always yields the second best solution: by never yielding the best solution it would be the worst in Fig. 7, while would be the second in Fig. 6. Additionally, it is interesting to note that the static version of BI, namely B, is much worse than many other competitors, including DEG and DEGI. This is probably due to the fact that the deletion of few nodes can substantially change the betweenness structure of the network, thus requiring an iterative computation; on the other hand, deleting one node can only change the degree of neighbouring ones by one.

### Comparing node rankings

As discussed in Section 3.1, competitors often compute different node rankings, thus yielding significantly different *R* values. An interesting question is the quantification of such difference: how much do the node rankings of two competitors coincide? Or, in other words, how different are the underlying node selection strategies? Note that this is not a trivial issue, as similar strategies, yielding slightly different node rankings, can result in radical different *R* values; similarly, two completely different strategies, i.e. exploiting different structural characteristics, may end up with compatible rankings.

**Figure 8:**
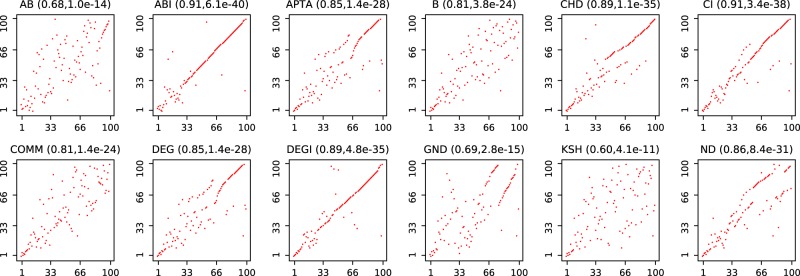
Correlation of node removal strategies for each competitor against BI, for a BA network with n = 100 and m = 3. For each pair of competitors, a dot represents a node in the network, while the x-coordinate is the rank in the first competitors and the y-coordinate is the rank in BI. A straight line indicates a perfect correlation between rank orders, while a scattered collection of points indicates rather uncorrelated ranks. For each pair of competitors, we show the correlation coefficient and p-value in parenthesis. For instance, ABI and BI are highly correlated, with a correlation coefficient of 0.91 and p-value of 6.1*e*^−40^ for this specific network.

In Fig. 8, we give an example for the node ranking similarity of all competitors against BI for a lesmis network. The more points are clustered around the main diagonal, the more correlated the two competitors’ rankings are. It is worth highlighting that ABI and BI are highly correlated, as expected being the former an approximated version of the latter. Additionally, all methods seem to converge to a common sequence in the final phase of an attack, indicating that the competitive advantage of BI resides in the initial choices. With this example in mind, we proceed to report some more general insights on node ranking similarity.

**Figure 9:**
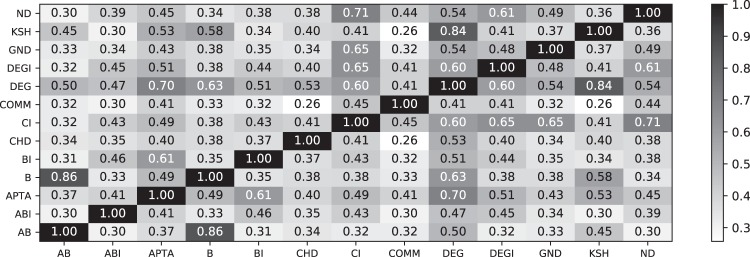
Order comparison of all against all for small values of R (smaller than 0.05). For each network realization, we have computed the pairwise correlation coefficient for all competitors. The number reports the median correlation coefficient over all network realizations. Cells filled in darker color have a larger median correlation coefficient. The diagonal represents pairs of identical competitors. B and AB are correlated most. Other interesting high correlations include CI/ND, DEG/KSH, and B/DEG.

In Fig. 9 we report the median correlation coefficient between node rankings obtained by all pairs of algorithms. This has been calculated considering all synthetic networks with *R* ≤ 0.05, the rationale being that we are interested in the node ranking similarity for highly vulnerable networks only. We can identify several pairs of highly-similar competitors. While B and AB are highly correlated, it is interesting to note that ABI and BI are not. This is a surprising result, and indicates that, while ABI is an approximated version of BI, they reach quite different solutions - a feature that does not happen in the B - AB case. Additionally, the apparently conflicting results of Figs 8 and 9 suggest a high variability, with correlated rankings only for some specific topologies. These results are further surprising if one considers that both ABI and BI compute very efficient attacks (as reported in Fig. 7). If two good solutions can be reached through two different paths, it may be expected a further improvement when both are combined - a relevant topic for future research. Figure 9 further suggests that DEG is more similar to any other method than the median of all other pairs; the degree sequence may thus be the best representative combination of all methods.

### Ranking for scalability

**Figure 10:**
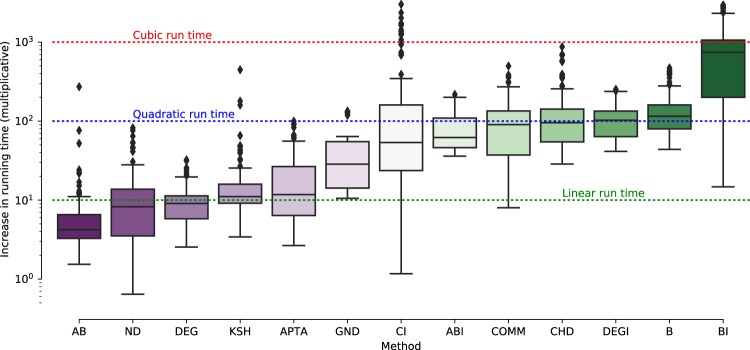
Scalability of competitors with an increasing number of nodes in the network. We report the running time increase for a network, once the number of nodes is increased by a factor of 10. For some competitors we observe a very large variation of running times (indicated by larger boxplots).

We analyze and compare the scalability of all competitors, an aspect of utmost importance in real applications. Towards this aim, we considered instances of the same network type, respectively with 100 and 1000 nodes, for then calculating the ratio between the median running time for both groups. Such ratio indicates how much the computational cost increases when the network size is one order of magnitude larger. The results are shown in Fig. 10. We find that around half of the methods (AB, ND, DEG, KSH, APTA, and GND) are rather scalable, with an almost linear increase of the computational cost. The worst case is observed for BI, with an increase of the run time by a factor of 10^3^, i.e. its computational cost scales with the cube of the number of nodes, preventing it to be executed even on medium-size networks. It is interesting to note the large spread of CI values: while being very efficient for tree-like and hierarchical structures, where nodes have a small (constant) maximum degree, CI becomes inefficient for networks with high degree nodes, since even small ball sizes require to repeatedly analyze large parts of the network.

### Optimality of methods regarding Pareto front

The results in Section 3.1 and Section 3.3 treat efficacy (in terms of obtaining small R values) and scalabiliy (increase in computational cost) as separate indicators. Nevertheless real experiments usually impose a trade-off between both aspects, as the size of the network and the limited availability of computational resources can force the choice of faster, if less accurate, methods. In what follows, we combine both aspects by analyzing the shape of the corresponding Pareto fronts.

**Figure 11:**
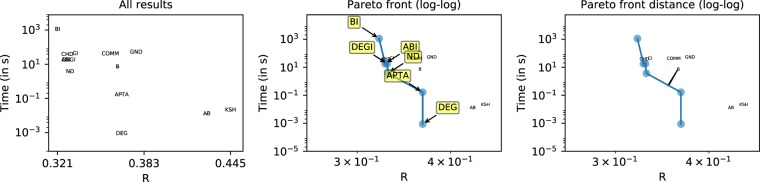
Visualization of Pareto front (center) and normalized Pareto front distance (right). Only few competitors, in this case five, contribute to the set of interesting solutions, which are identified by the Pareto front spanned up by two dimensions (R value and running time). BI is the most accurate competitor and also the slowest competitor. DEG, on the other hand, estimates the robustness very quick, but less accurate. ABI, DEGI, and ND provide a trade-off between fast execution and accurate R values. The y-axis (running time) is log-scaled. The normalized Pareto front makes the results comparable over different networks.

For the sake of clarity, Fig. 11 firstly illustrates the methodology with a specific example. It can be seen that out of 13 competitors, only five compute an interesting, non-dominated solution, while the remaining 8 are dominated in one of the two considered dimensions. In order to assess the quality of a solution, we measure the distance between the solution and the Pareto front - as in Fig. 11(right). The larger the distance, the less competitive is that specific solution. Moreover, we can measure the one-dimensional distance to the next best competitor on the Pareto front; depending on the direction, we can assess its competitiveness regarding quality and run time. Note that we measure the distance in the log-space, such that a distance of one means that we need to improve either the quality or the run time by one order of magnitude.

**Figure 12:**
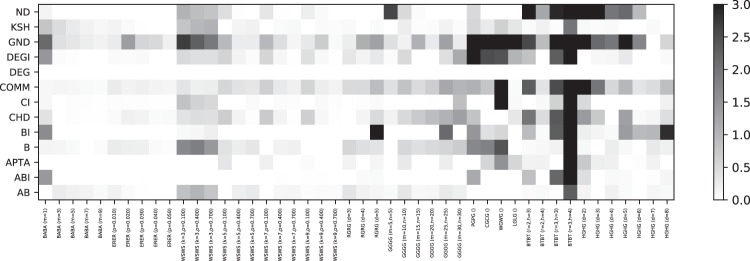
Distance from Pareto front for each competitor, broken down into network subtypes. A distance of zero (white color) indicates that a method is on the Pareto front. Larger distances are generally less interesting, since such competitors are dominated by other competitors. BI, DEG, and ABI often can be found on the pareto front and, therefore, provide non-dominating solutions. We can see that some methods are complementary in their distance to the Pareto front, for instance, ABI and AB.

**Figure 13:**
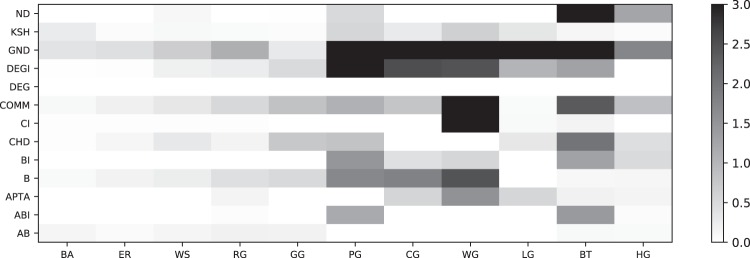
Distance from Pareto front for each competitor, aggregated by network types. Larger distances are generally less interesting, since such competitors are dominated by other competitors. Several networks stand out, e.g. WG, PG and BT, in that many competitors do not perform well on them, i.e., the Pareto front is much smaller. For instance, in case of BT, the degree of a node is sufficient to deduce a very effective node removal strategy. Most networks are dismantled by DEG and ABI in an effective way.

**Figure 14:**
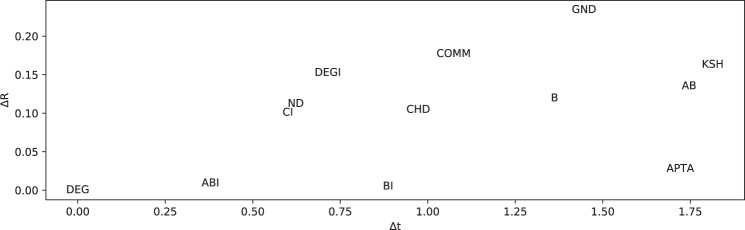
Pareto front-distance broken down into two dimensions: Inaccurary (ΔR) and slowness(Δt). The position of a competitor in a scatter plot reveals, in which direction a competitor should be improved, in order to be more competitive. For instance, BI is always very close to the Pareto front regarding the quality of solutions (small ΔR), but the running time should be reduced significantly (larger Δt). ND, on the other hand, should be improved to compute better attacks, in order to be more competitive.

In Fig. 12, we plot the median distance to the Pareto front for each competitor and network type. We can see that DEG is always on the Pareto front, because it computes the fastest attack. Similarly, BI and ABI are often on the Pareto front, because they compute very strong attacks and good trade-offs, respectively. Other competitors are close to the Pareto front for selected network types. A few competitors have rather large median distances, including B, which means that they are not very interesting from a quality nor a run time point of view. A further aggregated version of these results is shown in Fig. 13. In Fig. 14, we conclude how Pareto fronts indicate the goodness of a specific method and how it could be improved. Note that DEG is at the origin because it is the fastest competitor for each network in our study, and therefore, always on the Pareto front.

### Real-world networks

We finally complement the results obtained for the synthetic set with similar analyses for real-world networks. We applied all competitors to the following twenty real-world networks, used in many other studies on this subject, most of which can be downloaded from the UCI Network Data Repository^58^. The networks are listed in Table 2 along with selected topological properties.

**Table 2:** Overview of twenty real networks and selected network properties, ordered by the number of nodes.

Network	N	E	AVG Degree	Density	Assortativity
karate	34	78	2.29	0.13904	−0.476
dolphins	62	159	2.56	0.08408	−0.044
lesmis	77	254	3.30	0.08681	−0.165
polbooks	105	441	4.20	0.08077	−0.128
adjnoun	112	425	3.79	0.06837	−0.129
football	115	613	5.33	0.09352	0.162
celegansneural	297	2148	7.23	0.04887	−0.163
usair	332	2126	6.40	0.03869	−0.208
netscience	379	914	2.41	0.01276	−0.082
polblogs	1,222	16,714	13.68	0.02240	−0.221
petster-hamster	2,000	16,098	8.05	0.00805	0.023
facebook	4,039	88,234	21.85	0.01082	0.064
eva	4,475	4,652	1.04	0.00046	−0.185
power	4,941	6,594	1.33	0.00054	0.003
hep-th	5,835	13,815	2.37	0.00081	0.185
astroph	17,903	196,972	11.00	0.00123	0.201
condmat	21,363	91,286	4.27	0.00040	0.125
internet	22,963	48,436	2.11	0.00018	−0.198
enron	33,696	180,811	5.37	0.00032	−0.116
twitter	81,306	1,342,296	16.51	0.00041	−0.039

Values are for the giant component of the network.

**Figure 15:**
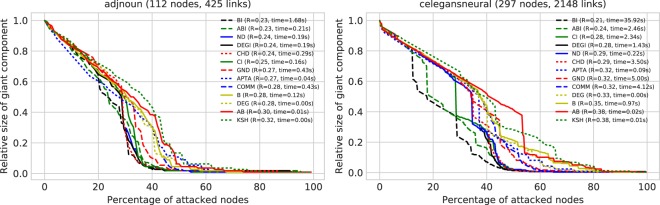
Visualization of the robustness curves for two real-world networks: Adjnoun and celegansneural.

**Figure 16:**
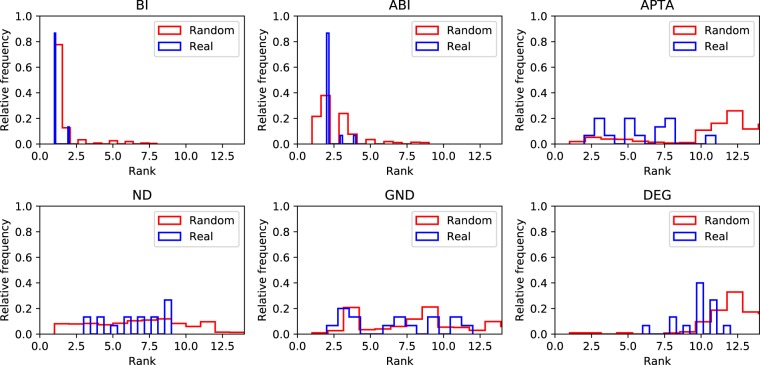
Comparison of ranking distributions for several real-world networks and the results obtained from random networks.

As an illustrative example, in Fig. 15 we visualize the robustness curves for the adjnoun and celegansneural networks - competitors are ranked in each figure according to the respective *R* value. It can be seen that results obtained for synthetic networks, in terms of ranking of methods according to their attack efficacy, match quite well with the real case. To further support this observation, we show the competitor ranking distribution according to the computation of the smallest R value in Fig. 16. We see that the ranking distributions between random and real-world networks largely coincide: results obtained for synthetic networks thus seem to be of general applicability.

### Other methods for measuring the robustness

**Figure 17:**
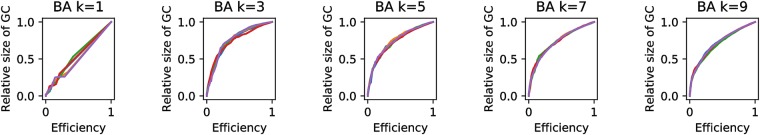
Comparison of the size of giant component with global efficiency.

In our previous experiments, we have analyzed the robustness of networks regarding R, which measures the relative size of the giant component during an attack process. Another measure for estimating the response of complex networks to disruptions is the global efficiency, which takes into account how efficiently information is propagated in a network^59^. The propagation efficiency between a pair of nodes is inversely proportional to shortest distance between both nodes in the network. In Fig. 17, we compare the global efficiency with the relative size of the giant component over time for some BA network instances with different *k*. Obviously, these two measures are highly correlated. In additional experiments, we compared the area under the curve (essentially the R value) for both measures and each network: The mean correlation coefficient over all networks is 0.943, i.e., both robustness measures are indeed highly correlated. Therefore, we believe that our choice of R over the relative size of the giant component is representative for a wider range of robustness measures.

### Sensitivity of COM to network modularity

**Figure 18:**
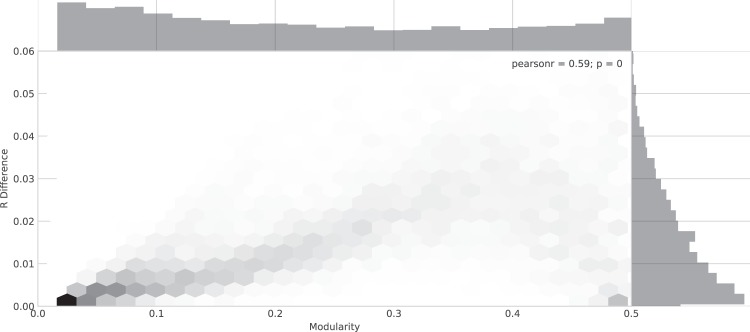
Effectivity of COM for networks with different modularity Y-axis is the difference of R values obtained by COM and those obtained by BI.

We have performed additional experiments where we compare the R values of COM with the best method, BI. The results are reported in Fig. 18. We find that for networks with very small modularity COM performs similar to BI, given that these networks are usually very robust to targeted attacks. With increasing modularity, the BI significantly outperforms COM, given the existence of vulnerable nodes. Only for networks with large modularity, COM become almost as effective as BI again, given that it can exploit community structure for attack generation. It should be noted that for our generated networks, COM never significantly outperforms BI, independent of the modularity.

## Conclusions

The problem of measuring the resilience of a network to targeted attacks, and the other side of the coin, i.e. the design of optimised strategies for network dismantling, are rapidly becoming major topics of research in the complex network community. This is undeniably due to the adverse consequences associated with a low resilience of real-world systems, and especially of critical infrastructures. As a consequence, many research works have appeared, and a large set of different attack strategies have been proposed. Yet, on the other hand, these proposals have not been organically organised: a practitioner would then find it difficult to compare them, and eventually choose the one most suitable for the system under analysis. In this contribution we have systematically evaluated the state-of-the-art in complex network robustness and dismantling. Specifically, a large set of algorithms have been compared, both against real and synthetic networks, and taking into account elements like different network sizes, link densities, and generation parameters. All strategies have been evaluated according to their accuracy, computational cost, and reliability. This yielded several important conclusions, that are discussed below.

First of all, our analysis allows identifying which are the best algorithms. It might be surprising to see that the best algorithm is BI (betweenness interactive), probably one of the oldest and conceptually simplest approaches. BI is efficient because the definition of the betweenness is extremely well aligned with the problem at hand - i.e. disrupt the main communication paths of the network. Employing BI comes at a price: A run time at least quadratic in the number of nodes, for sparse networks; and cubic in dense networks. Therefore, we need additional approaches for larger networks.

CI was published essentially comparing to degree-based attacks only, without a comparison to betweenness-based competitors (B or BI). The major contribution of CI regarding network dismantling is that it brought the idea of analyzing real-word network robustness into the top journals. CI was the first method to run in almost linear time, O(NlogN), for specific networks, and to significantly outperform degree in their experiments. Moreover, CI was specifically designed to find optimal attacks in hierarchical networks with tree structures. Many real-world networks, however, are not purely tree-structured. Therefore, it is not surprising to see that CI does not perform well, when running it on previously unseen network types and comparing it to previously unconsidered competitors. Regarding ND, it should first be noted that ND performs much better than CI in our study. Intuitively, this makes sense, since CI was the main competitor evaluated when ND was proposed and published. In addition, the authors of ND compared against degree, eigenvector centrality and a solution based on simulated annealing. Yet, the authors did not assess their method compared to betweenness or approximate versions of betweenness (B, BI, AB, and ABI), the strongest competitors in our study. So, similar to the case of CI, the performance of ND can be explained by the experimental setup of the authors. Many recently-proposed strategies are designed to provide suboptimal but computationally efficient solutions. The user should understand a tradeoff between effectiveness and speed. We see this as a major motivation and major contribution of our study, to compare a whole set of heuristics (including computationally expensive ones) on a wide range of methods, in order to assess the possible degree of optimality.

As a result of our study, we highly recommend the usage of BI for assessing the quality of novel proposed competitors on smaller datasets. Moreover, ABI should be the first choice as a competitor for larger networks, as it provides the best tradeoff between solution quality and runtime in our study. In order to foster the interpretability of results, and consequently increase the usefulness of this line of research, standards for the publication of new strategies should be raised. We believe that the present work can be a first small step in this direction.

In the present study, we have chosen the size of networks in such a way that all methods can be compared with reasonable runtime. According to our experiments, BI is the best method for all networks, but its runtime complexity is about *O*(*N*^3^). This implies that, if the size of an input network is increased by a factor of 10, the runtime will be approximately 1000 times longer. Given that we aimed at a full comparison of network methods, the execution of all experiments on hundreds of random networks is simply not feasible. Assessing the robustness of very large random networks with a reduced number of scalable methods, as identified in our study, is one possibility for future work.

Our study leads to several additional observations and interesting directions for future work. First, several methods are highly competitive with BI. The major goal for future research should be to make them more efficient regarding the run time. One challenging problem is to always maintain a list of connected components during an attack generation, while avoiding quadratic time complexity. Second, the use of approximate methods, such as ABI, for network dismantling should be explored and exploited further. Finally, we showed that ND and BI actually compute quite different attacks, while both being close to BI. An intelligent combination of these two methods might lead to even stronger attacks, possibly outperforming BI. Future studies could investigate the robustness of multiplex/multi-layer networks^60–63^.

## Data Availability

All networks used in this study are available from the UCI Network Data Repository^58^.
